# Mechanisms of hamstring strain injuries in sprinting: an integrative scoping review of intrinsic, biomechanical and modifiable risk factors

**DOI:** 10.3389/fphys.2026.1943228

**Published:** 2026-09-30

**Authors:** Junshuai Zhu, Shuaiwang Huang, Shuyuan Luo, Yelin Zeng, Lan Zheng, Xiyang Peng

**Affiliations:** 1 Key Laboratory of Physical Fitness and Exercise Rehabilitation of Hunan Province, College of Physical Education, Hunan Normal University, Changsha, China; 2 School of Sport and Health Sciences, University of Lancashire, Preston, United Kingdom

**Keywords:** biomechanics, hamstring strain injury, intrinsic muscle structures, risk factors, sprinting

## Abstract

This scoping review aimed to bring together evidence from muscular architecture, biomechanics, training physiology and injury history-fields that have been examined individually in the past-to clarify how intrinsic and extrinsic factors influence hamstring strain injury development. A systematic literature search of three databases was undertaken in accordance with the PRISMA guidelines for scoping reviews. Peer-reviewed studies covering the mechanisms of hamstring strain injuries were reviewed and 32 articles met the eligibility criteria and methodological quality assessment. Multiple intrinsic/outside risk factors for Hamstring strain injuries (HSIs) were identified in this review. The biceps femoris long head is at risk with eccentric loading due to the narrow width of the aponeurosis and stress applied through previous injury/biomechanical overload during late swing/late stance. Anterior pelvic tilt, trunk flexion, hip flexion, lower leg angular velocity, add to the risk of injury especially where previous injuries caused architectural/motor control changes. Factors that can be changed such as targeting resistance training can decrease the risk for HSIs while suddenly stopping training or muscle fatigue increases risk via increased muscle stiffness and the likelihood of reinjury. This scoping review compiles multi-disciplined evidence to support the argument that the hamstrings do not form a unitary structure.

**Systematic Review Registration:**

https://osf.io/, identifier afjek.

## Introduction

Hamstring strain injuries (HSIs) are among the most prevalent non-contact injuries in sport, accounting for 12-16% of all musculoskeletal injuries and with a recurrence rate of 22-34% (Opar et al., 2012; Martin et al., 2022; Alonso et al., 2012). Despite decades of targeted prevention and rehabilitation efforts, the incidence of HSIs has remained persistently high, with longitudinal surveillance data showing no significant decline over recent decades (Ekstrand et al., 2022). HSIs commonly occur in sports involving sprinting, acceleration, deceleration, rapid changes of direction, jumping, and stretching (Kelly et al., 2022), and are broadly categorized into high-speed running type and stretching type (Wing and Bishop, 2020). The present review focuses exclusively on the high-speed running type. The muscles most susceptible to injury, specifically the long head of the biceps femoris (BFlh), semitendinosus (ST), and semimembranosus (SM), may be affected in the muscle belly, the musculotendinous unit, or the tendon region. Recovery time varies by injury location (Opar et al., 2012). The reported incidence of HSIs is 24% in football, 17% in track and field, and 22% in rugby (Chen et al., 2018; Augustsson and Andersson, 2023; Alonso et al., 2012), resulting not only in impaired athletic performance and reduced training and competition time but also in substantial economic burden in team sports (Hamilton, 2012; Ripley et al., 2021).

Acute HSIs may result from direct external impact, traumatic events, overuse (exceeding the muscle’s load capacity), or secondary injury. Chronic injuries typically arise from diminished muscle function due to inadequate healing or rehabilitation after a prior injury (Huygaerts et al., 2020). Severity is classified into three grades: grade I involves damage to a small number of muscle fibers with minor functional loss; grade II involves significant damage without complete rupture, accompanied by substantial functional loss; and grade III is characterized by complete muscle fiber rupture and total loss of function (Larson et al., 2024).

### Applied anatomy and biomechanics

The hamstring muscles are located on the posterior thigh and comprise the biceps femoris (long head, BFlh; short head, BFsh), semimembranosus (SM), and semitendinosus (ST). The BFlh, SM, and ST are biarticular muscles spanning the hip and knee, functioning as both hip extensors and knee flexors (Huygaerts et al., 2021). Additionally, they assist in internal and external rotation of the leg during knee flexion (Bramah et al., 2024; Chu and Rho, 2016). The BFlh, SM, and ST originate from the ischial tuberosity; however, some evidence suggests the BFlh also attaches to the sacroiliac joint (SIJ) (Huygaerts et al., 2020), implicating it in pelvic stability and suggesting that SIJ or pelvic alterations may influence BFlh stress distribution. The BFlh is the most frequently injured muscle during high-speed running (Kelly et al., 2022; Wing and Bishop, 2020; Kerin et al., 2023), particularly during the late swing and early stance phases, where it experiences extreme peak forces. During the stance phase, the ST serves as the primary load-bearing muscle, whereas the SM is more vulnerable in stretch-type injuries (Wing and Bishop, 2020). The BFsh, the only monoarticular hamstring muscle, originates from the lateral lip of the linea aspera and functions primarily in knee flexion with slight external rotation. Both BFlh and BFsh attach to the lateral fibular head; additionally, the BFlh has been reported to attach to the lateral tibial condyle and lateral collateral ligament (Huygaerts et al., 2021). The ST inserts on the medial aspect of the tibial tuberosity, while the SM inserts partly on the posteromedial tibial condyle, partly fuses with the popliteal fascia, and partly reinforces the oblique popliteal ligament of the knee capsule (Pimenta et al., 2018). These distal connections contribute to knee joint stability during rotation and translation (Ernlund and Vieira, 2017).

The connection between the internal muscle architecture and the inherent risks associated with HSIs is still being examined. For example, Guanche (2015) proposed that the innervation of the biceps femoris by both the tibial nerve branch to BFlh and peroneal nerve branch to the BFsh may create a vulnerability to tensile failure from uncoordinated contractions between the two heads, although this has yet to be confirmed (Huygaerts et al., 2021). In addition, several sources have indicated that the disproportionately high proportion of type II (fast-glycolytic) muscle fibers in hamstrings may create greater susceptibility to injury from eccentric contractions as supported by several animal studies (Wing and Bishop, 2020). However, the clinical relevance of these findings is still uncertain because an individual’s fiber type is often influenced by age and training history.

### Hamstring biomechanics

During sprinting, which is the activity most strongly associated with HSIs, the hamstrings are subjected to substantial mechanical demands, particularly during acceleration and maximal-velocity phases (Nakamura et al., 2016). In the acceleration phase, the horizontal component of the ground reaction force is a key determinant of performance (Vicens-Bordas et al., 2024), and the hamstrings generate substantial hip extension torque, contributing approximately 15% of total propulsive force (Martín-Ruiz et al., 2024). During maximal-velocity sprinting, peak hamstring forces reach 3 to 4.2 times body weight during stance and up to 8 times body weight during the late swing phase (Brusco et al., 2023). The running gait cycle is typically divided into stance (foot in contact with the ground) and swing (foot not in contact) phases, with further subdivisions including the pre-stance (braking), post-stance (propulsion), pre-swing (reduction), and post-swing (pre-activation) phases (**Figure 1**) (Bramah et al., 2024; Danielsson et al., 2020). During the swing phase, the hamstrings must resist knee extension torque; during stance, they must counteract external joint torques generated by ground reaction forces, including hip flexion and knee extension torques (Sun et al., 2015). As running speed and cumulative exposure increase, so do the magnitude of muscle forces, neural activation, and muscle stretch, all of which elevate injury risk (Sun et al., 2015; Yu et al., 2017).

**Figure 1:**
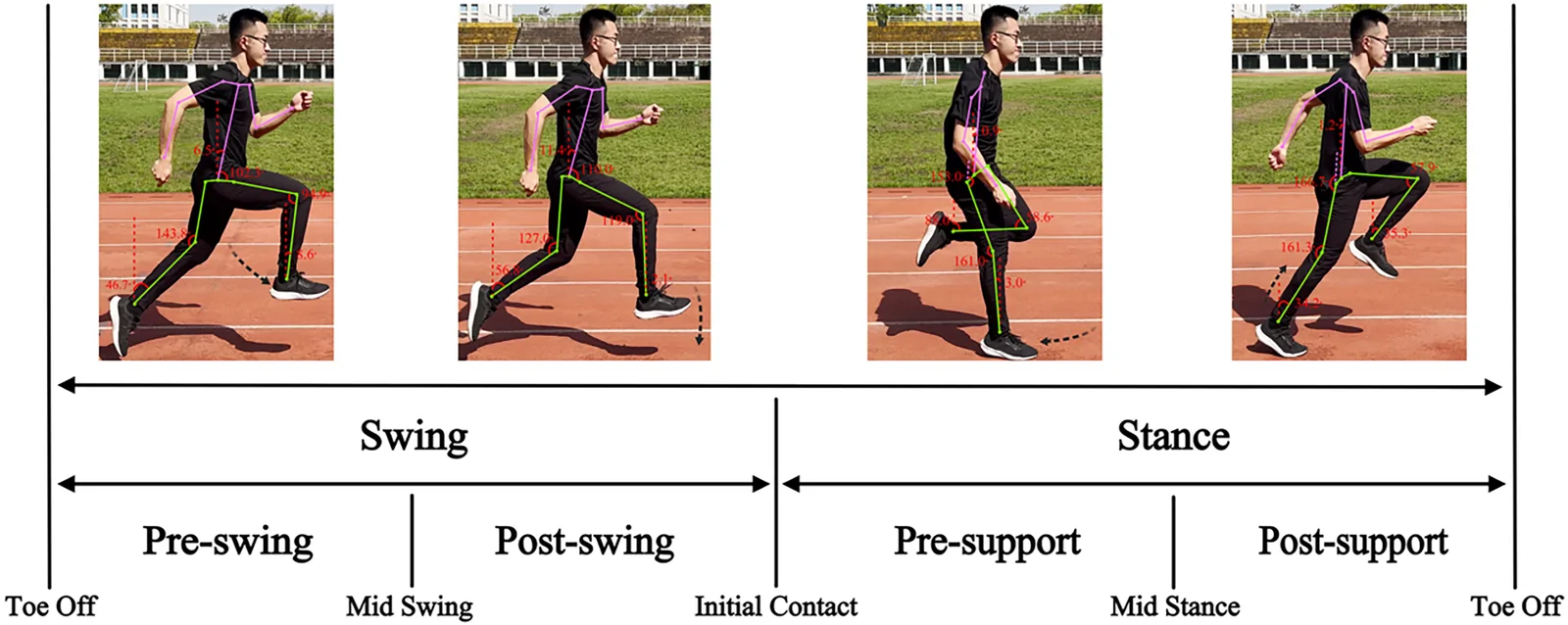
The running gait cycle. The gait cycle is divided into swing and stance phases, with further subdivisions including pre-swing, post-swing, pre-support (braking), and post-support (propulsion) phases. Key events such as toe-off, mid-swing, initial contact, and mid-stance are indicated. The late swing and early stance phases, during which the hamstrings experience peak mechanical loads, are highlighted as periods of elevated injury risk. The pre-swing, mid-swing, and late swing phases are distinguished in the left panel by the corresponding limb positions, with the late swing phase representing the terminal portion of swing when the hamstrings reach their greatest length and eccentric loading.

Given the hamstrings’ pelvic origin, excessive forward trunk lean can further destabilize the pelvis by placing it in an anteriorly tilted position, increase hip flexor torque, and alter hamstring tissue length during hip rotation (Svensson et al., 2016). Repetitive loading under such conditions leads to cumulative metabolic and mechanical fatigue, exacerbating microtrauma and predisposing to HSIs (Wing and Bishop, 2020; Bramah et al., 2024; Brusco et al., 2023). Lumbar hyperextension may serve as a compensatory response to anterior pelvic tilt. Some case reports and spine-focused studies suggest that subtle nerve compression or nerve root irritation resulting from lumbar extension could alter motor neuron function and increase HSIs risk, although robust evidence supporting this association is lacking (Freckleton and Pizzari, 2013).

### Other mechanisms

Beyond anatomical and biomechanical factors, several additional mechanisms contribute to HSIs risk. Flexibility and strength are inversely correlated with peak tendon stress; targeted flexibility training combined with eccentric strengthening effectively reduces tendon stress (Freckleton and Pizzari, 2013; Askling et al., 2006). Similarly, stretching has been shown to lower peak muscle stress and ground reaction forces (Ruan et al., 2018). Athletes with a history of HSIs exhibit reduced optimal muscle stretch length, tendon thickening that increases local stress, and sagittal-plane hip asymmetry (Silder et al., 2010a). Under fatigue, the hamstrings demonstrate diminished capacity for energy absorption and dissipation, while the quadriceps assume a greater load-sharing role, thereby increasing strain vulnerability (Evangelidis et al., 2023). Following just two weeks of training cessation, hamstring stiffness increases, and eccentric strength declines significantly, raising injury risk (Yamashita et al., 2023; Miyamoto et al., 2024). Neuromuscular control and inter-muscular coordination play a critical role in modulating hamstring strain risk. The hamstring-to-quadriceps (H: Q) strength ratio has been widely used as an indicator of knee joint muscle balance, with a low ratio being associated with increased injury risk (Evangelidis et al., 2015). Croisier et al. (2008) reported that soccer players with significant strength imbalances exceeding 15% had a 4- to 5-fold increased risk of hamstring injury, and correcting these imbalances to below 5% substantially reduced injury risk. However, the predictive value of the H:Q ratio remains debated, as some prospective studies have failed to confirm its utility (van Dyk et al., 2016). The concept of synergistic dominance describes a compensatory mechanism whereby impaired gluteus maximus function increases workload on the hamstrings, thereby elevating injury risk (Iguchi et al., 2023). Schuermans et al. (2017) found that lower gluteus maximus activity during the swing phase was associated with a higher risk of hamstring injury. Together, these findings underscore the importance of assessing proximal musculature and inter-muscular coordination when evaluating HSIs risk and designing prevention strategies.

Given the persistent high incidence and recurrence of HSIs despite existing prevention efforts, a comprehensive synthesis of the multifactorial mechanisms underlying HSIs in sprinting is warranted. This systematic scoping review aims to synthesize the available evidence on intrinsic and extrinsic risk factors, with the goal of informing more effective prevention and rehabilitation strategies.

## Materials and methods

### Study design

Given the heterogeneity in study designs, populations, and outcome measures across the existing literature on HSIs mechanisms, a scoping review framework was chosen to map the breadth of evidence and identify key knowledge gaps (Arksey and O'Malley, 2005; Tricco et al., 2018). While scoping reviews do not always require formal quality assessment, we additionally evaluated methodological rigor to ensure the reliability of included studies. The review was conducted in accordance with the Preferred Reporting Items for Systematic Reviews and Meta-Analyses extension for Scoping Reviews (PRISMA-ScR) (Tricco et al., 2018).

### Search strategy

In October 2025, two authors conducted a systematic search of three databases (PubMed, Web of Science and Cochrane Library). There were no restrictions on the publication year. Search terms were as follows **Table 1**.

**Table 1:** Search strategy.

Database	Search strategy
PubMed	((((“hamstring strain injuries”[Mesh] OR “hamstring strain injuries”[Title/Abstract]) AND (mechanisms[Title/Abstract] OR etiology[Title/Abstract] OR risk factors[Title/Abstract]) AND (“biceps femoris”[Title/Abstract] OR “semitendinosus”[Title/Abstract] OR “semimembranosus”[Title/Abstract]) AND (“sprinter”[Title/Abstract] OR “track and field athlete”[Title/Abstract] OR “running”[Mesh]) AND (“run”[Title/Abstract] OR “sprint”[Title/Abstract] OR “acceleration”[Title/Abstract]))))
Web of Science	(TS=(“hamstring strain injuries” OR “hamstring strain”) AND TS=(mechanism* OR etiology OR “risk factor*”) AND TS=(“biceps femoris” OR semitendinosus OR semimembranosus) AND TS=(sprinter* OR “track and field athlete*” OR running) AND TS=(run OR sprint* OR acceleration))
Cochrane library	(hamstring* NEAR/3 (strain* OR injur*)) AND (mechanism* OR etiology OR “risk factor*”) AND (“biceps femoris” OR semitendinosus OR semimembranosus) AND (sprinter* OR “track and field” OR running) AND (run OR sprint* OR acceleration)

### Study selection criteria

#### Inclusion criteria

(1) Study Type: Experimental, observational, or modeling-based original research; (2) Participants: Adults (≥18 years) who were athletes or physically active individuals (i.e. engaged in regular structured sport or exercise training), with or without a history of hamstring injuries, particularly athletes; (3) Research Focus: Investigations centered on hamstring morphology, biomechanics, injury mechanisms, or training interventions; (4) Data Metrics: Quantitative measurements including hamstring length, strain, stiffness, strength, or activation patterns; (5) Publication Type: Peer-reviewed articles published in English-language journals.

#### Exclusion criteria

(1) Study types: Review, case report, animal study, conference abstract; (2) Study participants: Minors (<18 years) or physically inactive populations, defined as individuals not engaged in regular structured physical activity or sport (e.g. <150 minutes of moderate-intensity or <75 minutes of vigorous-intensity activity per week, as determined by the original study’s reported activity levels); (3) Research focus: Not primarily targeting hamstring muscles or solely focusing on surgical interventions; (4) Data reporting: Unclear methodological descriptions or inability to extract quantitative data; (5) Publication type: Non-English papers or inaccessible full-text articles.

### Data extraction

The data for each study was extracted by the lead author (JZ). Specifically, for each included study, we extracted data on the following predefined items to align with PRISMA-ScR guidelines: (1) study design, (2) sample size and demographics, (3) specific muscle investigated (BFlh/ST/SM), (4) measurement tools (MRI/US/EMG), (5) running phase analyzed (swing/stance), (6) key biomechanical parameters, (7) main conclusions on injury risk. After the initial data extraction was completed, a portion of the research samples was randomly selected and handed over to another author (SH) for verification to ensure the accuracy of the extracted data. Disagreements between reviewers regarding study eligibility or data extraction were resolved through discussion; if consensus could not be reached, a third reviewer (SL) was consulted for final arbitration. This approach ensured the reliability of the collected information and reduced errors or discrepancies in the dataset.

### Quality assessment

Given the methodological heterogeneity of the included studies (ranging from observational cohort designs to cadaveric dissections and computational simulations), we developed a standardized quality appraisal tool tailored to the specific demands of biomechanical and physiological injury-mechanism research. This tool was informed by established reporting guidelines, including the STROBE Statement for observational studies (Vandenbroucke et al., 2007) and the TREND Statement for non-randomized interventions (Des Jarlais et al., 2004), and adapted to accommodate laboratory-based biomechanical investigations. The ten-item criteria encompass: (1) clarity of research questions and hypotheses; (2) appropriateness of participants and sampling; (3) rigor of experimental design; (4) reliability of measurement methods and instruments; (5) adequacy of data processing and statistical analysis; (6) completeness of results presentation; (7) depth of result interpretation and discussion; (8) ethical compliance and transparency; (9) ecological and external validity; (10) practical value and translational significance. The included articles were evaluated by two independent reviewers in accordance with the predefined scoring criteria. In cases where consensus was not reached between the two reviewers, a third reviewer was consulted to resolve discrepancies.

Two independent reviewers (JZ and SH) piloted the tool on three randomly selected studies to calibrate scoring consistency prior to full assessment. Discrepancies were resolved through discussion; if consensus could not be reached, a third reviewer (XP) arbitrated. The final scores were categorized as high quality (≥ 9/10), moderate quality (7-8/10), or low quality (≤ 6/10). These thresholds were established *a priori* based on team consensus after pilot testing on three randomly selected studies. No study was excluded based on quality score; rather, the scores were used to contextualize the strength of the evidence in the narrative synthesis.

## Results

### Search results

An initial database search yielded 5,190 articles, and an additional 128 articles were obtained through other channels. After removing duplicates (1,673 articles) and irrelevant articles (2,953 articles), 692 articles were retained for the abstract screening process. The result of the abstract screening was that 228 full-text articles remained after excluding 464 articles. Another 196 articles were excluded due to not meeting the inclusion/exclusion criteria, and ultimately 32 articles were included in the scope assessment study (**Figure 2**).

**Figure 2:**
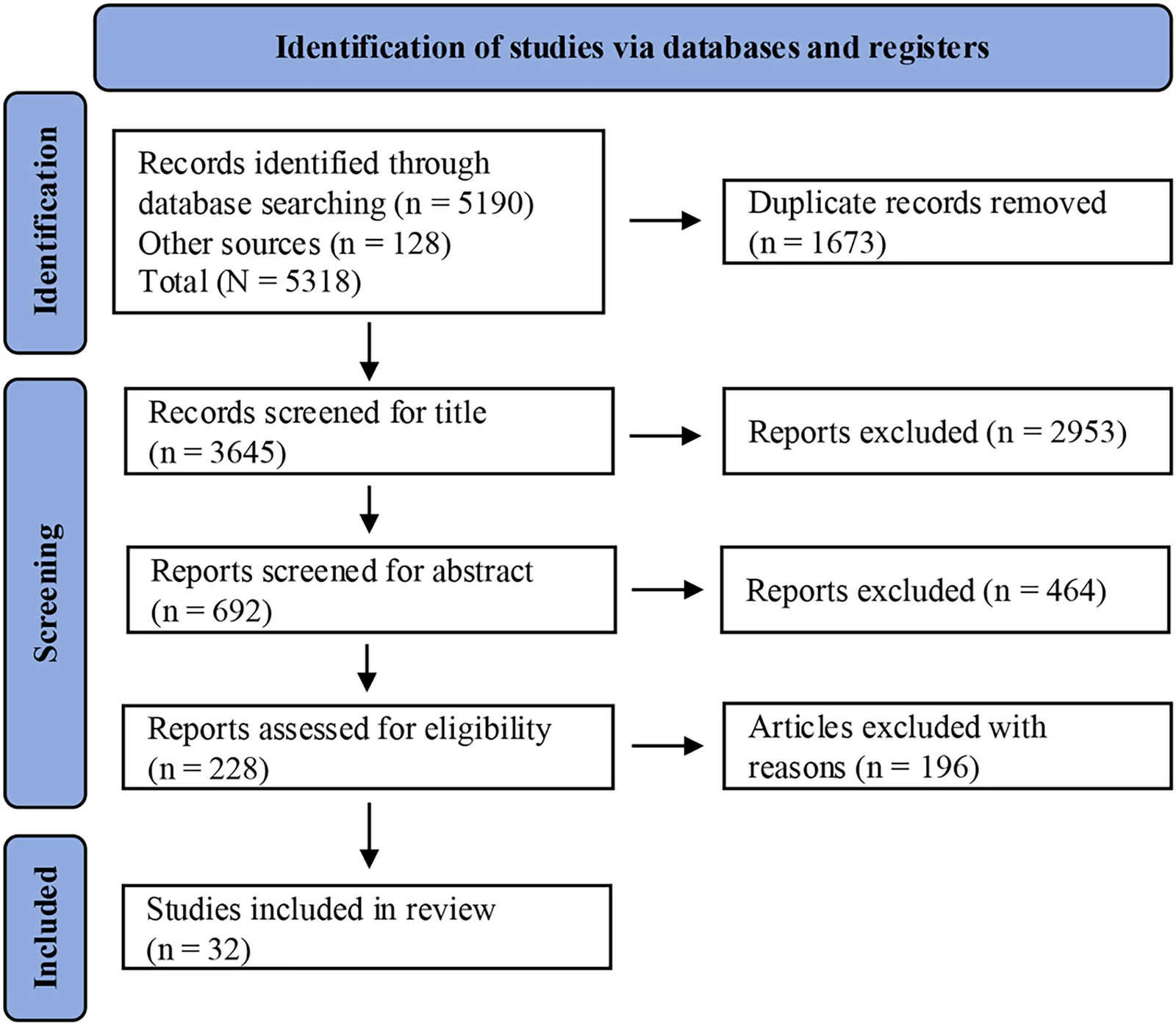
PRISMA (Preferred Reporting Items for Systematic Reviews and Meta-Analyses) flowchart illustrating the step-by-step process leading to identification of studies eligible for the review.

Among the 32 included studies, 6 investigated the influence of internal muscle structure on hamstring strain (Fiorentino et al., 2012, Fiorentino and Blemker, 2014; Lazarczuk et al., 2024; Storey et al., 2016; Miyamoto and Hirata, 2021; Evangelidis et al., 2015), 13 studies explored the pathogenic mechanism related to biomechanical factors (Mendiguchia et al., 2021, Mendiguchia et al., 2024; Guex et al., 2012; Kellis et al., 2024; Emami et al., 2020; Chumanov et al., 2011; Zhong et al., 2017; Schache et al., 2012; Sahinis and Kellis, 2024; Gurchiek et al., 2024; Morin et al., 2015; Cerone et al., 2023; Hegyi et al., 2019), and the remaining 13 studies examined other factors including flexibility and strength, stretching, previous injury history, fatigue and training arrangement (Ruan et al., 2018; Silder et al., 2010a; Evangelidis et al., 2023; Yamashita et al., 2023; Miyamoto et al., 2024; Wan et al., 2017a, 2017b; Wan et al., 2021; Guex et al., 2016; Proske et al., 2004; Tokutake et al., 2018; Daly et al., 2016; Silder et al., 2010a). The number of participants included in the study ranged from 1 to 61 (a total of 507 participants), with ages ranging from 18 to 30 years old. Seven studies analyzed the injury conditions of individuals with a history of hamstring strain (Silder et al., 2010a, 2010b; Lazarczuk et al., 2024; Kellis et al., 2024; Emami et al., 2020; Proske et al., 2004; Daly et al., 2016), among which five compared the cases of previous injuries with those of uninjured samples (Lazarczuk et al., 2024; Kellis et al., 2024; Emami et al., 2020; Proske et al., 2004; Silder et al., 2010b). Two studies dissected cadaveric thighs and analyzed the characteristics of tendon injuries (Storey et al., 2016; Mendiguchia et al., 2024). A total of 23 studies analyzed uninjured individuals and evaluated the mechanism of hamstring injuries (Ruan et al., 2018; Evangelidis et al., 2023; Yamashita et al., 2023; Miyamoto et al., 2024; Fiorentino et al., 2012; Fiorentino and Blemker, 2014; Miyamoto and Hirata, 2021; Evangelidis et al., 2015; Mendiguchia et al., 2021; Guex et al., 2012; Guex et al., 2016; Chumanov et al., 2011; Zhong et al., 2017; Schache et al., 2012; Sahinis and Kellis, 2024; Gurchiek et al., 2024; Morin et al., 2015; Cerone et al., 2023; Hegyi et al., 2019; Wan et al., 2017a, 2017b; Wan et al., 2021; Tokutake et al., 2018).

### Quality assessment

The methodological quality scores for the 32 included studies are summarized and visualized in the narrative breakdown below. The overall quality was moderately high: 24 studies were rated as high quality (≥ 9/10), 6 studies as moderate quality (7-8/10), and 2 studies as low quality (≤ 6/10). Of the six studies examining intrinsic muscle structure (**Table 2**), five scored ≥ 9, except for Storey et al. (2016) (scoring 8/10), primarily due to its reliance on a small cadaveric sample (n=5) which limited generalizability. In the biomechanics domain (**Table 3**), the two cadaveric studies (Mendiguchia et al., 2024) and EMG-based studies (Cerone et al., 2023) received relatively lower scores (7-8/10) due to deductions in ecological validity and sample size justification. Among the studies on other contributing factors (**Table 4**), the two training-cessation studies (Yamashita et al., 2023; Miyamoto et al., 2024) scored 7 and 9 respectively, with the former losing points for inadequate control of pre-intervention training loads. Notably, no study was excluded based on quality assessment. Instead, lower-scored studies were interpreted with greater caution in the discussion, and their findings are primarily used to generate hypotheses rather than to draw definitive conclusions.

**Table 2:** Pathogenic mechanisms of intrinsic muscle structures.

Authors	Subjects & methods	Aim/purpose	Results & conclusions	Total score
Fiorentino et al. (2012)	13 (5 female, 8 male) healthy people (mean age: 25 ± 5 years, height: 175 ± 7 cm) Aponeurosis width assessment, dynamic imaging methods	To assess the impact of muscle activation and structure on live muscle function and strain injury risk	The proximal aponeurosis of the BFlh femoris exhibits a relatively narrow structure and is subjected to substantial tensile stresses	9
Lazarczuk et al. (2024)	26 recreationally active males with and without prior unilateral HSI within the past 24 months MRI, statistical parametric mapping	Comparing BFlh geometry and proximal aponeurosis characteristics between injured and uninjured limbs	The injured limb exhibited reduced muscle-to-fascia volume ratio and increased proximal BFlh fascial volume versus the uninjured control limb	10
Storey et al. (2016)	10 thighs from 5 male cadavers versus 20 thighs from 10 active young men Morphometric analysis, MRI and MTJs	Through the anatomical analysis of the MTJ, the relevant factors leading to hamstring strain were identified	Certain factors reduce the pressure at the proximal connection of the SM, making the BFlh the most vulnerable site for proximal hamstring strain	9
Fiorentino and Blemker (2014)	12 University of Virginia track and field athletes (8 male) MRI, Finite element meshes, and Simulation boundary conditions	Quantifying the size and tissue strain of the BFlh during high-speed running through computational modeling	The relative dimensions between the aponeurosis and muscle influence the internal strain	9
Miyamoto and Hirata (2021)	16 men (173 cm, 68 kg, 23 years) participated Ultrasound elastography, B-mode US	Determine the changes in the active muscle stiffness along the long axis and the tension of the proximal aponeurosis of the BFlh during its contraction	BFlh muscle stiffness was greater at the distal end at low intensities, not moderate-to-high, and no strain patterns were noted in the proximal aponeurosis across intensities	10
Evangelidis et al. (2015)	30 healthy recreationally active participants (age, 20.7 ± 2.6 yr; height, 1.79 ± 0.07 m; body mass, 72.2 ± 7.2 kg) MRI, Max voluntary contractions for knee flexion	To examine correlations between the proximal aponeurosis area of the BFlh and its ACSAmax, as well as isometric and eccentric knee flexor strength	Aponeurosis size in young men varies, unrelated to muscle size or knee flexor strength, and a smaller aponeurosis may increase the risk of hamstring injuries	9

*MRI*, Magnetic Resonance Imaging; *MTJ*, musculotendinous junction; *ACSAmax*, maximal cross-sectional area.

**Table 3:** Biomechanical pathogenic mechanisms.

Authors	Subjects & methods	Aim/purpose	Results & conclusions	Total score
Mendiguchia et al. (2022)	15 amateur men athletes (1.79 ± 0.75 m, 77.0 ± 7.6 kg) 3D sprinting was assessed	Evaluate the impact of an intervention integrating lumbopelvic control and running technique on sprint kinematics and performance	Six-week multimodal training altered sprinting kinematics, potentially reducing muscle strain risk and improving performance	10
Mendiguchia et al. (2024)	7 fresh-frozen specimens (full lower limb with pelvis and lumbar spine) Specimen dissection, three-dimensional reconstruction system	Anterior pelvic tilt leads to hamstring elongation, with greater proximal than distal muscle lengthening	Excessive pelvic anteversion creates disproportionate tension in the BFlh, ST, and SM, with pronounced elongation of the SM proximally	7
Guex et al. (2012)	10 elite sprinters (5 male, 5 female; age = 21.2 ± 3.6 years, height = 175 ± 6 cm, weight = 63.8 ± 9.9 kg). Muscle contractions with one leg at different angles (0°, 30°, 60° and 90°flexion)	The influence of hip flexion angle on the strength ratio of hamstrings to quadriceps	Hamstring torque is lowest at 0° hip flexion and highest at 90°, especially during eccentric contraction. The hamstring-to-quadriceps ratio increases with greater hip flexion	8
Kellis et al. (2024)	13 male soccer players lacking hamstring injury history versus 11 with prior hamstring injury experience. SWE, EMG	To examine the differences in TLF and lumbar muscle stiffness in injured versus uninjured subjects via SWE	SWE modulus of the TLF, ES, and MF was higher in soccer players with a history of hamstring injury compared to their uninjured counterparts	9
Emami et al. (2020)	15 male soccer players (ages 18-30), HSI-present and absent Two American exploratory devices	Consistency of ultrasound-measured abdominal and lumbar multifidus thickness in athletes with and without hamstring injuries	Intraclass correlation coefficients for athletes showed high reliability for abdominal muscle thickness and MF muscle CSA, with values of 0.82-0.93	10
Chumanov et al. (2011)	12 subjects (9 males, 3 females) volunteered to participate in this study (age: 24.5 ± 4.1 yrs, height: 176.1 ± 5.0 cm, mass: 70.2 ± 8.8 kg) EMG, Treadmill, Whole-body musculoskeletal model and Muscle control algorithm	To compare hamstring musculotendon stretch, loading, and work done during stance and swing phases of high-speed running using forward dynamic simulations	In the gait cycle’s 50-90% (swing phase), hamstrings load and shorten. This loading increases with speed, reaching a peak at faster gaits when swing phase loading surpasses stance phase loading	8
Zhong et al. (2017)	8 national-level male sprinters 3D kinematics data, GRF and the intersegmental dynamics method	Quantifying lower extremity joint torques and muscle group power contributions	Hamstring muscles face increased stress in initial stance and late swing phases due to ground reaction forces and lower limb movement	9
Schache et al. (2012)	7 subjects (5 males and 2 females) 3D model and GRF	Hamstring injury mechanics during sprinting inform vulnerability to stretch-related damage	Hamstring injuries peak during terminal swing, exhibiting maximum musculotendon force and strain in BF, ST, and SM	9
Sahinis and Kellis (2024)	Fifteen moderately active males (22.1 ± 0.3 years; 89.1 ± 3.9 kg; 1.86 ± 0.05 m) averaging 1870 ± 800 MET-minutes/week. 3D ultrasound images	It remains uncertain if BFlh exhibits distinct mechanical properties compared to other hamstring muscles	BFlh rigidity and stress increase muscle fascicle energy absorption over the distal tendon in the sprint’s late swing phase	9
Gurchiek et al. (2024)	10 participants (5 female, 5 male) Open Cap, 3D musculoskeletal model, linear mixed models, and running speed as covariate	Comparing hamstring dimensions and velocities during accelerating and steady-speed runs	Hamstrings are subjected to longer lengths and faster lengthening velocities in accelerative running than in constant-speed running	8
Morin et al. (2015)	14 male subjects (mean ± SD; body mass: 79.9 ± 7.9 kg; height 1.79 ± 0.07 m; age 24.2 ± 4.6 years) Treadmill sprint kinetics and EMG	The role of the hip extensors in horizontal GRF production during maximal, treadmill sprint accelerations	Maximum horizontal force production correlated with both high pre-contact hamstring activation and elevated eccentric hamstring peak torque	9
Cerone et al. (2023)	10 male athletes (range: 19–31 years; 59–83 kg; 1.68–1.98 m) Sprinters performed 70%, 85%, and 100% maximal efforts on an 80-m track	The distribution of excitation within muscles in running at different speeds changes	EMG amplitude rose with running speed during the late swing and early stance phases, particularly in the proximal thigh region (2-10% of thigh length) at 70-100% of max speed	8
Hegyi et al. (2019)	13 male participants (mean ± SD: age, 25 ± 3 yr; height, 1.80 ± 0.07 m; body mass, 79 ± 9 kg; preferred kicking leg, 3 left, 10 right); EMG	To examine the proximal–distal and intermuscular activity of BFlh and ST as increasing running speed	EMG activity increased uniformly across all bilateral muscle regions during late swing as running speed escalated, peaking in all regions	9

*SWE*, Shear wave elastometry; *TLF*, thoracolumbar fascia; *ES*, erector spinae; *MF*, multifidus; *EMG*, Electromyography; *GRF*, ground reaction force.

**Table 4:** Other pathogenic mechanisms.

Authors	Subjects & methods	Aim/purpose	Results & conclusions	Total score
Wan et al. (2017a)	10 male and 10 female undergraduates 3D kinematics, Sprinting kinematics	To examine the relationship between hamstring flexibility and peak hamstring muscle strains in sprinting	Hamstring strains are frequent in sprinting, tied to lower flexibility, with strain severity varying among muscles, affecting injury rates	9
Wan et al. (2017b)	21 university students (11 male, 10 female) Athlete’s hamstring elasticity, Isokinetic muscle force and the 3D motion patterns	Investigating correlations between optimal hamstring muscle length, flexibility, and strength	The greater the flexibility of the hamstring muscles, the longer the optimal stretching range. Compared with men of the same flexibility, women usually have a shorter optimal stretching range	10
Wan et al. (2021)	20 male college students (aged 18–24 years) Paired t-tests	To determine the effects of altering hamstring flexibility or strength on peak hamstring musculotendinous strain during sprinting	Participants in the flexibility intervention group showed increased SM and BFlh lengths. The strength group significantly enhanced hamstrings length and decreased sprinting strain	9
Guex et al. (2016)	20 national-level sprinters (7 men, 13 women; age range, 16–26 years; height range, 161.5–186.5; body mass range, 48.5–76.6 kg) Tests for isokinetic and flexibility	To determine whether eccentric hamstring training during the preparatory phase can enhance the strength, strength ratio, optimal angle and flexibility of elite sprinters	Improving sprinters’ routines with increased eccentric hamstring training may reduce risks associated with hamstring injuries, addressing strength balance, muscle ratio, and flexibility	10
Ruan et al. (2018)	12 female college athletes (age: 20.8 ± 0.7 years; height: 1.61 ± 0.05 m; body mass: 54.25 ± 4.22 kg) 3D kinematic data and EMG	Evaluating the influence of hamstring stretching on sprint injury risk by analyzing its effect on the length-torque dynamics	Hamstring strengthening induced rightward shifts in torque curves for knee and hip joints during late swing and early stance phases, potentially reducing hamstring strain risk	10
Proske et al. (2004)	9 athletes with a history of unilateral hamstring strains and 18 athletes with no previous history of strain injuries Optimal torque angles were measured	Identifying athletes at risk of hamstring strains and how to protect them	The angle at which the previously injured muscle achieved its maximum torque was shorter than that of the corresponding muscle in the uninjured leg and the muscles in the uninjured group	9
Tokutake et al. (2018)	61 male track and field athletes (age, 19.6 ± 1.1 years; 95% confidence interval, 19.3-19.9) Testing ROM and Questionnaire	Investigating the association between HSIs during rapid running and the structure and function of hip muscles	The injured athletes have a significantly higher risk of injury and a greater range of passive hip joint motion than the uninjured athletes	10
Silder et al. (2010a)	18 subjects with a previous hamstring injury (>5 months prior) MRI, Isokinetic strength testing and Treadmill sprinting	Conducting on the data changes of sprinting for athletes with unilateral HSIs to evaluate the association with scar tissue formation	Persistent scar tissue may follow acute muscle strain injuries, yet these morphological alterations typically do not manifest at the joint level mechanically or in neuromuscular control patterns	8
Daly et al. (2016)	9 elite male Gaelic games athletes Lower limb muscle kinematics and activities	Investigate rehabilitating HSIs through analysis of EMG and kinematic traits in previously injured athletes	Injured athletes showed lower biceps femoris activation in late swing relative to other muscles, with hip, pelvic, and knee asymmetries	10
Silder et al. (2010b)	11 healthy and 8 subjects with prior biceps femoris injuries MRI and Phase-contrast cine imaging	To evaluate the impact of a history of HSIs on tissue displacement and strain during active stretching contractions	Dynamic contractions enhance biceps femoris injury risk, and post-injury tissue responses deviate from usual patterns	9
Evangelidis et al. (2023)	21.8-year-old; height 172.9 cm, weight 67.2 kg; iPAQ 1768 MET-mins/week (mean ± SD) MVT, SWV and Transverse relaxation times	Fatigue on hamstrings active muscle stiffness and contraction type effects during submaximal fatiguing tasks	Hamstring muscles fatigue differently during eccentric contractions, with BFlh showing more sensitivity, suggesting increased injury likelihood	8
Yamashita et al. (2023)	13 young male highly trained sprinters (average World Athletics points = 978) Isokinetic knee moments	Sprinters’ knee extension and flexion torque reduction following two weeks of training cessation	After two weeks break, sprinters regenerate explosive power through fast fibers while building controlled eccentric strength for knee movement	7
Miyamoto et al. (2024)	28 healthy male track-and-field sprinters (20.0 ± 1.2 years, 173.7 ± 5.6 cm, 65.6 ± 6.6 kg) Ultrasound elastography	BFlh passive stiffness changes after 2 weeks training cessation in sprinters	Muscles stiffen by training cessation in sprinting athletes	9

*EMG*, Electromyography; *ROM*, range of motion; *MRI*, Magnetic Resonance Imaging; *SWE*, Shear wave elastometr.

### Pathogenic mechanisms of intrinsic muscle structures

**Table 2** summarizes the characteristics, methods, and key findings of the six studies that examined the influence of intrinsic muscle structure on HSIs (Fiorentino et al., 2012, Fiorentino and Blemker, 2014; Lazarczuk et al., 2024; Storey et al., 2016; Miyamoto and Hirata, 2021; Evangelidis et al., 2015). Four studies used MRI, static imaging, or dynamic imaging to measure the proximal aponeurosis width of the BFlh, ST, and SM (Fiorentino et al., 2012, Fiorentino and Blemker, 2014; Lazarczuk et al., 2024; Evangelidis et al., 2015).

When the BFlh is actively stretched, its proximal narrow tendon produces relatively large amounts of displacement which increase the likelihood of tendon injury (Fiorentino et al., 2012; Evangelidis et al., 2015). Injured tendons in comparison to non-injured have been reported to be significantly decreased in terms of the volume of muscle versus aponeurosis (Lazarczuk et al., 2024). The BFlh tendon in previously injured limbs exhibited significantly greater thickness than in uninjured contralateral limbs which may have resulted in changing the local stress distribution on the tendon. In addition, decreased width of the aponeurosis combined with an increased volume of muscle has been shown to increase the risk of tendon injury, with higher levels of strain being present at the proximal myotendinous junction (Fiorentino and Blemker, 2014).

A cadaveric dissection study comparing male specimens with normal thighs revealed that the SM has the longest tendon and myotendinous junction (MTJ), making it susceptible to stretching injuries (Storey et al., 2016). Although the ST has the largest ratio of muscle belly volume to tendon interface area, other anatomical factors may offset force concentration at its proximal tendon junction, rendering the BFlh more vulnerable to proximal hamstring strain (Storey et al., 2016).

In contrast, one study using ultrasound shear wave elastography to measure shear wave velocity (a surrogate for stiffness) at proximal, central, and distal BFlh sites during 20%, 50%, and 80% of maximal voluntary isometric contraction (MVC) in knee flexion, along with B-mode ultrasound to assess regional elongation of the proximal BFlh tendon, found that muscle stiffness was greater in the distal than in the proximal BFlh at low intensities, but not at medium or high intensities. Furthermore, no site-specific feature of aponeurotic strain was observed proximal to the BFlh during contraction (Miyamoto and Hirata, 2021).

### Biomechanical pathogenic mechanisms

**Table 3** summarizes the characteristics, methods, and main findings of the 13 included studies investigating biomechanical mechanisms of HSIs. Ten studies involved sprinters (Mendiguchia et al., 2021; Guex et al., 2012; Chumanov et al., 2011; Zhong et al., 2017; Schache et al., 2012; Sahinis and Kellis, 2024), two involved soccer players (Kellis et al., 2024; Emami et al., 2020), and one used cadaveric sample (Mendiguchia et al., 2024). Participants included recreational and competitive athletes aged 18–30 years, with sample sizes ranging from 7 to 26.

A six-week multimodal training intervention reduced muscle strain risk by improving pelvic and lower limb kinematics: decreased pelvic tilt angle, higher vertical knee position of the forward swing leg, increased thigh angular velocity and retraction velocity, shorter touchdown time, and reduced leg distance before touchdown (Mendiguchia et al., 2021). Cadaveric dissection combined with 3D reconstruction revealed that increased pelvic anteversion angle caused a significant and uneven length increase in all three hamstring muscles, particularly the SM (Mendiguchia et al., 2024), underscoring the role of pelvic position in modulating hamstring tension.

A larger hip flexion angle, indicated by a higher vertical knee position, was associated with greater hamstring torque at 90°compared with 30°and 60°, increasing strain probability (Guex et al., 2012). Using shear wave elastography and ultrasound, two studies found that football players with a history of HSIs had thicker trunk muscles, including the thoracolumbar fascia, lumbar muscles, and abdominal muscles (Kellis et al., 2024; Emami et al., 2020).

Three studies using whole-body kinematics, EMG, ground reaction forces, and 3D ultrasonography reported greater hamstring loading during the swing phase, predisposing to strain (Chumanov et al., 2011; Schache et al., 2012; Sahinis and Kellis, 2024). However, one study noted substantial passive torsional loads during the initial stance phase, suggesting that both phases contribute to injury risk (Zhong et al., 2017).

Two studies analyzed the acceleration phase and found that muscle stretch length and velocity were greater than during maximal-velocity running, with higher negative peak torque during horizontal force production (Gurchiek et al., 2024; Morin et al., 2015). EMG activity increased progressively with running speed, peaking at the end of the swing phase (Hegyi et al., 2019). Additionally, BFlh EMG activity at the end of swing, measured in the distal region (2–10% of thigh length), increased when running speed rose from 70% to 100% of maximum, further linking BFlh injury risk to running speed (Cerone et al., 2023).

### Other pathogenic mechanisms

**Table 4** summarizes the characteristics, methodologies, and key findings of the included studies. Four articles examined the influence of flexibility and strength on HSIs using 3D kinematics, MRI, exercise training, and other methods (Wan et al., 2017a, 2017b; Wan et al., 2021; Guex et al., 2016). Peak hamstring strain was negatively correlated with flexibility, with greater strain in the BFlh and ST than in the SM, and no gender difference was observed (Wan et al., 2017a). Flexibility and strength training increased optimal hamstring tendon length and reduced peak tendon strain during running, although optimal muscle length was shorter in women than in men (Wan et al., 2017b; Wan et al, 2021). Maintaining a favorable tendon-to-muscle ratio and flexibility, combined with eccentric strengthening targeting the swing phase, effectively reduced strain risk (Guex et al., 2016). Static stretching significantly reduced peak horizontal and vertical ground reaction forces and BFlh activation during the terminal swing phase, suggesting a biomechanically plausible reduction in HSIs risk, though direct evidence of injury prevention remains to be established (Ruan et al., 2018).

Five articles investigated the impact of prior hamstring injury on HSIs risk using MRI, isokinetic strength testing, biomechanical assessment, and questionnaires (Silder et al., 2010a, Silder et al., 2010b; Proske et al., 2004; Tokutake et al., 2018; Daly et al., 2016). Prior injury was associated with a high reinjury rate (Tokutake et al., 2018). Athletes with a previous strain exhibited shorter optimal muscle lengths at peak torque (Proske et al., 2004), reduced pre-activation of the BFlh relative to the ipsilateral gluteus maximus, psoas major, and external oblique muscles during terminal swing, and marked sagittal-plane asymmetries in hip and pelvic kinematics (Daly et al., 2016). Active lengthening contractions induced locally elevated tissue stresses, with the proximal BFlh tendon being particularly vulnerable (Silder et al., 2010b). Although injured-side BFlh tendon volume was increased, no differences were observed in strength, maximal muscle stretch amplitude, or activation patterns (Silder et al., 2010a). Shear wave velocity measurements demonstrated that the BFlh fatigues more readily than the ST or SM during eccentric contractions, offering insight into its higher injury susceptibility (Evangelidis et al., 2023).

Two different studies investigated the effect of a variety of training regimens on HSIs (Yamashita et al., 2023; Miyamoto et al., 2024), both having similar study design, i.e. two weeks of specialized training vs control. Post-training, both groups of athletes reported reduced capacity to perform high-intensity rapid concentric contractions, decreased slow-eccentric strength and increased stiffness which all may increase the risk of future HSIs.

## Discussion

The current scoping review integrates the existing literature across the broad disciplines familiar to human performance and training, specifically: muscular architecture; biomechanics of sport; physiology of training; and history of injury. This interdisciplinary methodology allows for the development of a comprehensive, multi-dimensional framework of understanding the mechanisms of pathogenesis for hamstring strain injuries during sprinting (**Figure 3**).

**Figure 3:**
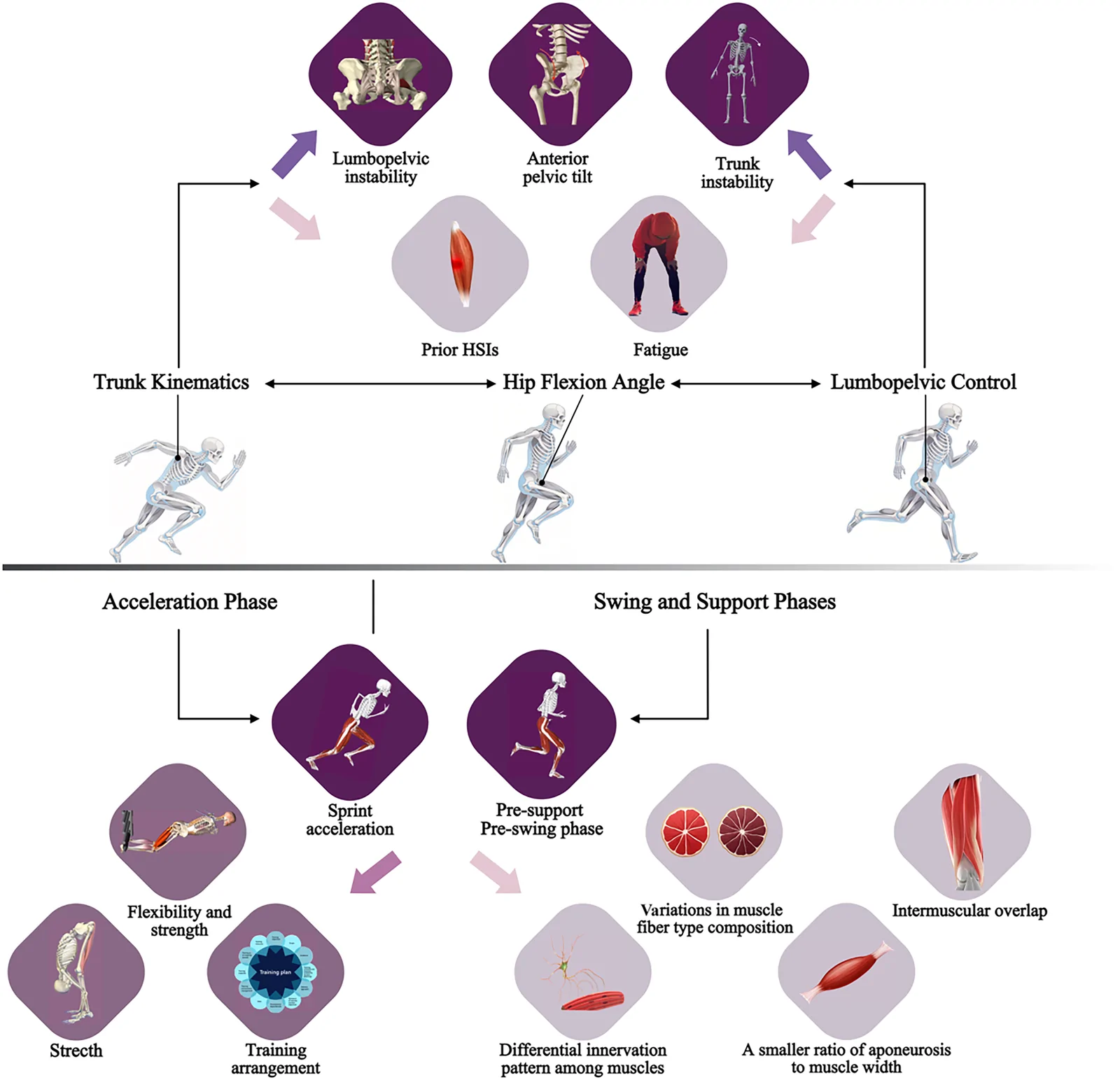
An integrative framework that demonstrates how intrinsic, biomechanical, and contributory factors interact in hamstring strain injuries while sprinting. This framework expands the analytical view of isolated muscle-tendon unit loading to include the coordinated functioning of the entire kinetic chain, as opposed to only looking at hamstrings as a single unit. The three domains interact with one another dynamically. That is, intrinsic muscle structure mechanisms (for example, reduced aponeurosis-to-muscle width ratio, different muscle fibre type composition, intermuscular overlap) interact with biomechanical mechanisms (for example, lumbopelvic instability, anterior pelvic tilt, trunk instability, altered trunk kinematics, hip flexion angle, acceleration factors, pre-support phase factors, and pre-swing phase factors) and other contributory factors (for example, history of HSIs, fatigue, flexibility and strength, stretching and training method) and these interactions affect running biomechanics in all phases of sprinting, with modifiable factors representing potential intervention targets.

### Intrinsic muscle structure mechanisms

#### Muscle architecture and overlap

The biarticular muscles, specifically the BFlh, ST, and SM, are most susceptible to strain injuries. Shared origins and insertions with varying morphologies result in partial muscular overlap. Research indicates proximal and distal tendon overlap between the BFlh and SM, but not with ST, potentially increasing strain risk. Additionally, the downward extension and rotation of the SM tendon deviates the muscle direction from the tendon, predisposing to strain (Dolman et al., 2014). Future dynamic analysis of the muscle-tendon-bone complex is needed to determine whether individual muscle characteristics or inter-muscular interactions primarily drive injury risk (Schlink et al., 2022).

#### Structure-function relationships

The BFlh and SM generate greater force and stiffness during contraction due to shorter muscle fibers and larger pennation angles, whereas the BFsh and ST achieve a greater range of motion and higher contraction speed through longer muscle fibers and smaller pennation angles. These structural differences may increase strain susceptibility. Kawama et al. (2024) reported that during active stretching of the BFlh, proximal tendon displacement and local stress exceed those in the distal region. Storey et al. (2016) observed that distal muscle hardness surpasses proximal hardness at low intensities, with no regional differences at medium to high intensities. Although the latter study achieved a quality score of 9, its limited sample size precludes broad generalizability. The ratio of aponeurosis width to muscle width serves as a critical risk indicator, with multiple high-quality studies (scores 9 or 10) demonstrating that reduced aponeurosis width relative to muscle width elevates strain susceptibility (Huygaerts et al., 2021; Hassid et al., 2024).

#### Post-injury architectural alterations

When soft tissues sustain damage, the ratio of aponeurosis volume to muscle volume is considerably reduced when compared with healthy tissue. The decrease in aponeurosis strength (or thickening) may result in alterations to the local stress profile of the tissue, particularly during the pre-support, pre-swing and acceleration phases, potentially increasing risk for repetitive strain injuries (Lazarczuk et al., 2024; Silder et al., 2010b). While some prospective studies have examined the association of aponeurosis area with muscle size, the exact width ratio that predicts the likelihood of occurrence of HSIs has not yet been established and research regarding the thickness of tendons is also limited.

#### Muscle fiber type

Early hypotheses suggested that a high proportion of type II (fast-glycolytic) muscle fibers in the hamstrings contributes to injury risk due to rapid force generation and lower fatigue resistance (Wing and Bishop, 2020; Huygaerts et al., 2021). However, Evangelidis et al. (2017) found that BFlh fiber composition does not appear to explain its high strain incidence. Cadaveric research indicates significant regional variation in fiber type distribution within the same muscle (Garrett et al., 1984), which may influence regional stress and fatigue. Measuring muscle fibers remains highly invasive, limiting research in this area (Huygaerts et al., 2021; Heiderscheit et al., 2010; Grange et al., 2023).

This section provides evidence that the hamstrings should not be considered as a single homogeneous muscle group. Furthermore, the BFlh may be particularly susceptible to strain injury, as suggested by its specialized architectural and mechanical properties, including the identification of the aponeurosis-tomuscle proportions as a possible predictor of potential susceptibility to strain injury.

### Biomechanical mechanisms

#### Lumbopelvic control

Lumbopelvic control has been consistently associated with HSI risk in cross-sectional studies (Bramah et al., 2024; Tsaklis et al., 2015), although the causal nature of this relationship remains to be established. Anatomical connections between the hamstrings, ischial tuberosity, and thoracolumbar fascia facilitate force transmission during locomotion. Impaired lumbopelvic control disrupts this transmission, increasing hamstring stress and injury risk. Athletes with prior HSIs exhibit altered muscle thickness and activation patterns correlated with poor lumbopelvic control (Đorđević et al., 2022; Avrillon et al., 2020a). Anterior pelvic tilt, often resulting from muscular imbalance, causes asymmetric hamstring hypertrophy and elevated strain risk during running (Opar et al., 2014). Performing hip flexion and extension while the hamstrings are elongated significantly increases strain risk (Bramah et al., 2024; Kalema et al., 2021).

#### Trunk kinematics

The length-tension ratios of trunk muscles change with trunk inclination, flexion, and rotation, thereby affecting pelvic stability. The additional lengthening of hamstrings and increase in hip flexor torque caused by excessive forward trunk inclination increase the demand on hip extensors to prevent fatigue and microtrauma (Pietraszewski et al., 2021). There is a clinical correlation between forward trunk inclination and hamstring injury, but no reliable experimental evidence has been found establishing a causal relationship.

#### Hip flexion angle

A larger hip flexion angle optimizes sprint propulsion by providing greater swing clearance and higher angular velocity before ground contact (Avrillon et al., 2020b). However, hamstring torque peaks at 90 degrees of hip flexion, corresponding to the highest injury risk compared to 30 and 60 degrees (Guex et al., 2012). Mendiguchia et al. (2024) reported that hip flexion angle decreases following hamstring injury as a kinematic adaptation. From a whole-limb perspective, increased flexion angle is often accompanied by elevated lower leg angular velocity, warranting further investigation using comprehensive lower limb biomechanical models.

#### Swing and support phases

Three independent studies confirmed substantial hamstring loading during the swing phase (Chumanov et al., 2011; Schache et al., 2012; Sahinis and Kellis, 2024), while another identified significant stress during the terminal swing and initial stance phases (Zhong et al., 2017). Quality assessment scores ranged from 8 to 9, with deductions primarily for ecological validity. Ono et al. (2015) demonstrated that ST stress exceeds BFlh stress during swing, whereas BFlh stress is higher during early stance. Lateral hamstrings remain consistently active throughout the gait cycle, which may contribute to the BFlh’s heightened susceptibility observed in epidemiological studies.

The precise timing of hamstring strain injury during sprinting has been directly documented through motion capture of an actual injury event. Heiderscheit et al. (2005) analyzed the running kinematics of an athlete who sustained an acute BFlh injury during treadmill running and identified a 130 ms window during the late swing phase as the period of injury, during which the BFlh reached a peak musculotendon length 12% beyond its upright posture length. This case report provides the most direct evidence that the late swing phase is the critical window for HSI occurrence. The mechanical demand on the hamstrings during this window is further modulated by proximal synergists. Lewis and Sahrmann (2009) demonstrated that during prone hip extension, medial hamstrings are activated prior to the gluteus maximus, suggesting that the hamstrings may be preferentially recruited when gluteal function is suboptimal. This pattern is consistent with synergistic dominance, whereby impaired gluteus maximus function increases compensatory hamstring load and strain risk.

#### Acceleration phase

Throughout the acceleration phase of a sprint, increased muscle stretch and velocity than found during running with maximal velocity, result in much greater negative peak torque values and a corresponding increase in electromyographic activity that is progressively related to the increase in velocity (Takahashi et al., 2021; Martín-Ruiz et al., 2024). Proper knee, hip, and ankle extension along with correct forward direction of swing have been shown to be critical variables affecting performance, whereas incorrect foot strike positions, incorrect tibial angles, or improper trunk positions can have a significant impact on injury risk (Carragher et al., 2019; Oleksy et al., 2021; Malliaropoulos et al., 2012).

By expanding this segment’s focus beyond strictly examining the lower extremities’ functions to include additional global kinetic chain elements, such as lumbopelvic control, trunk kinematics, and hip flexion angles, these other global elements help to emphasize that injury risk to the muscle-tendon unit occurs during the running swing phase, but is related to the coordination by all of the kinetic chain through all phases of a sprint and therefore has an impact on the risk for injury.

### Other contributing mechanisms

#### Flexibility and stretching

Peak hamstring stress is inversely correlated with flexibility, with BFlh and ST showing greater levels than SM, and no gender differences observed (Wan et al., 2021; Tsaklis et al., 2015). Flexibility and eccentric training improve tendon length and reduce peak tendon stress (Guex et al., 2016; Oleksy et al., 2021; Askling et al., 2006). Although females have shorter optimal muscle lengths, no peak stress differences exist, highlighting the need for gender-specific investigations. Stretching reduces muscle stress and ground reaction forces, lowering HSIs risk, though static stretching may impair explosive power in high-intensity activities (Schmitt et al., 2012; Mulligan et al., 2025; Freckleton and Pizzari, 2013). Individual variability necessitates further research on optimized stretching protocols.

#### Prior injury

Athletes with prior strain have a shorter length of muscle than athletes without prior strain. They also exhibit less pre-activation in their terminal swing and greater asymmetry in their hip in the sagittal plane (Đorđević et al., 2022). The presence of previous strains causes tendon thickening and increased stress concentration and local sensitivity. Collectively, these cross-sectional findings suggest that athletes with previous strains may be at elevated risk for repeated injuries, although the observational nature of the available evidence precludes causal inference (Wing and Bishop, 2020; Danielsson et al., 2020; Takahashi et al., 2021).

#### Training and deconditioning

Hamstring stiffness and reduction of eccentric strength caused by erratic normalization of training schedule, increases risk of hamstring strains (Yamashita et al., 2023; Miyamoto et al., 2024). Coaches should keep track of muscle condition throughout the training cycle and especially at transitions between off-season and pre-season and at the end of the competitive season. Evaluating the strength of the hamstrings through data can help prevent HSIs. Studies that have looked at the effect of stopping training have generally had a short period of intervention and few have recorded their training schedule adequately; indicating a need for better methods and protocols.

This section identifies flexibility deficits, previous injury and training irregularities as modifiable risk factors that can be significantly reduced with targeted intervention to reduce HSIs risk when addressed. Therefore, these factors represent actionable targets for injury prevention.

The integrative framework developed in this review not only consolidates our understanding of HSIs pathogenesis but also delineates specific, mechanism-informed intervention targets. From an architectural perspective, the disproportionate vulnerability of the BFlh, driven by its narrow proximal aponeurosis relative to muscle volume, suggests that interventions should prioritize eccentric loading exercises that specifically target the BFlh at long muscle lengths such as lengthened-state eccentric training to promote favorable architectural adaptations, including increased fascicle length and aponeurosis width. From a biomechanical standpoint, the critical role of the late swing phase in injury occurrence, combined with evidence linking anterior pelvic tilt, trunk kinematics, and synergistic dominance to excessive hamstring loading, points to the need for interventions that address proximal control and inter-muscular coordination. Specifically, lumbopelvic stabilization training, gluteus maximus strengthening, and running technique modification (e.g. reducing excessive forward trunk lean and optimizing pelvic positioning) represent logical preventive strategies that directly counter the biomechanical drivers identified in this review. Furthermore, from a physiological perspective, the heightened reinjury risk following previous HSIs, together with the rapid deconditioning observed after training cessation, underscores the importance of structured, individualized return-to-sport protocols that monitor muscle stiffness and eccentric strength as objective indicators of tissue readiness. However, it is important to acknowledge that the current evidence base, while supportive of these mechanistic inferences, remains predominantly cross-sectional and observational; prospective intervention studies are urgently needed to validate whether targeting these specific mechanisms translates into clinically meaningful reductions in HSIs incidence. Future research should adopt standardized outcome measures and incorporate dose-response designs to establish optimal training volumes, intensities, and progression criteria for each identified intervention target.

## Limitations

Although this review offers a novel synthesis across disparate evidence domains, several methodological and inferential constraints must be acknowledged. Substantial heterogeneity in participant characteristics, measurement protocols, and outcome definitions across studies, compounded by predominantly small-sample cross-sectional designs, strictly limits our capacity to infer causality or quantify the dynamic interactions among identified factors, such as how altered aponeurosis morphology modifies biomechanical loading patterns. The predominance of laboratory-based assessments may not fully capture the ecological complexity of neuromuscular fatigue and decision-making during actual competition. Furthermore, the absence of standardized anatomical thresholds, for example aponeurosis-to-muscle width ratio, together with the invasive nature of fibre-type analyses, impedes direct translation into stratified clinical practice. Consequently, our integrative framework should be interpreted as hypothesis-generating rather than definitive, underscoring the urgent need for prospective longitudinal cohort studies with harmonized protocols to disentangle these intricate causal pathways.

Furthermore, while we conducted a formal quality appraisal using a standardized tool, the criteria and weighting were developed specifically for this review and have not been externally validated. Although pilot testing and dual-reviewer calibration were performed, the assignment of scores inevitably involves subjective judgments, particularly for items such as ecological validity and translational significance. Consequently, our quality stratification should be interpreted as an auxiliary heuristic for weighting evidence, rather than as a definitive measure of internal validity. Future research would benefit from establishing discipline-specific consensus tools for appraising biomechanical and physiological mechanism studies.

## Conclusions

This scoping review builds upon existing knowledge about hamstring strain injuries related to sprinting, by collating data from muscular architecture, biomechanics, training physiology and previous injury history. The results challenge the way that the hamstring muscles are viewed as one homogenous group, in that there is considerably more structural and functional variability that occurs within these muscles. From a biomechanical perspective, an individual’s overall risk of injury is not only attributable to an isolated loading of the muscle-tendon unit; rather, it stems from the overall coordination of the whole kinetic chain throughout all phases of sprinting. Previous injuries can continue to place individuals at risk through the chronic changes that occur within the tissue, while modifiable factors can be targeted for successful injury prevention strategies. The breadth of the evidence suggests that there is a need for well controlled, prospective studies to validate this integrated framework and provide guidance for individualized, stratified injury prevention strategies.

## Data Availability

The original contributions presented in the study are included in the article/**Supplementary Material**. Further inquiries can be directed to the corresponding author.
