# Supplementary material for: Mechanisms of hamstring strain injuries in sprinting: an integrative scoping review of intrinsic, biomechanical and modifiable risk factors
Source: Front Physiol. 2026 Sep 30;17:1943228. doi: 10.3389/fphys.2026.1943228 (PMC13661361; doi:10.3389/fphys.2026.1943228)
Supplement: Supplementary file 1 [file Table1.docx]

|  | 1 | 2 | 3 | 4 | 5 | 6 | 7 | 8 | 9 | 10 | Total Points |
| --- | --- | --- | --- | --- | --- | --- | --- | --- | --- | --- | --- |
| Fiorentino et al. (2012) | 1 | 1 | 1 | 0 | 1 | 1 | 1 | 1 | 1 | 1 | 9 |
| Lazarczuk et al. (2024) | 1 | 1 | 1 | 1 | 1 | 1 | 1 | 1 | 1 | 1 | 10 |
| Storey et al. (2016) | 1 | 0 | 1 | 1 | 1 | 1 | 1 | 1 | 1 | 1 | 9 |
| Fiorentino et al. (2014) | 1 | 1 | 1 | 1 | 0 | 1 | 1 | 1 | 1 | 1 | 9 |
| Miyamoto et al. (2021) | 1 | 1 | 1 | 1 | 1 | 1 | 1 | 1 | 1 | 1 | 10 |
| Evangelidis et al. (2015) | 1 | 1 | 1 | 0 | 1 | 1 | 1 | 1 | 1 | 1 | 9 |
| Mendiguchia et al. (2022) | 1 | 1 | 1 | 1 | 1 | 1 | 1 | 1 | 1 | 1 | 10 |
| Mendiguchia et al. (2024) | 1 | 0 | 0 | 1 | 1 | 1 | 1 | 1 | 0 | 1 | 7 |
| Guex et al. (2012) | 1 | 1 | 0 | 1 | 1 | 1 | 1 | 1 | 0 | 1 | 8 |
| Kellis et al. (2024) | 1 | 1 | 1 | 0 | 1 | 1 | 1 | 1 | 1 | 1 | 9 |
| Emami et al. (2020) | 1 | 1 | 1 | 1 | 1 | 1 | 1 | 1 | 1 | 1 | 10 |
| Chumanov et al. (2011) | 1 | 1 | 1 | 1 | 0 | 1 | 1 | 1 | 0 | 1 | 8 |
| Zhong et al. (2017) | 1 | 1 | 1 | 1 | 0 | 1 | 1 | 1 | 1 | 1 | 9 |
| Schache et al. (2012) | 1 | 1 | 1 | 1 | 0 | 1 | 1 | 1 | 1 | 1 | 9 |
| Sahinis et al. (2024) | 1 | 1 | 1 | 0 | 1 | 1 | 1 | 1 | 1 | 1 | 9 |
| Gurchiek et al. (2024) | 1 | 1 | 1 | 0 | 1 | 1 | 1 | 1 | 0 | 1 | 8 |
| Morin et al. (2015) | 1 | 1 | 1 | 0 | 1 | 1 | 1 | 1 | 1 | 1 | 9 |
| Cerone et al. (2023) | 1 | 1 | 0 | 1 | 1 | 1 | 1 | 1 | 0 | 1 | 8 |
| Hegyi et al. (2019) | 1 | 1 | 1 | 0 | 1 | 1 | 1 | 1 | 1 | 1 | 9 |
| Wan et al. (2017a) | 1 | 1 | 0 | 1 | 1 | 1 | 1 | 1 | 1 | 1 | 9 |
| Wan et al. (2017b) | 1 | 1 | 1 | 1 | 1 | 1 | 1 | 1 | 1 | 1 | 10 |
| Wan et al. (2021) | 1 | 1 | 0 | 1 | 1 | 1 | 1 | 1 | 1 | 1 | 9 |
| Guex et al. (2016) | 1 | 1 | 1 | 1 | 1 | 1 | 1 | 1 | 1 | 1 | 10 |
| Ruan et al. (2018) | 1 | 1 | 1 | 1 | 1 | 1 | 1 | 1 | 1 | 1 | 10 |
| Proske et al. (2004) | 1 | 1 | 1 | 1 | 0 | 1 | 1 | 1 | 1 | 1 | 9 |
| Tokutake et al. (2018) | 1 | 1 | 1 | 1 | 1 | 1 | 1 | 1 | 1 | 1 | 10 |
| Silder et al. (2010a) | 1 | 1 | 0 | 0 | 1 | 1 | 1 | 1 | 1 | 1 | 8 |
| Daly et al. (2016) | 1 | 1 | 1 | 1 | 1 | 1 | 1 | 1 | 1 | 1 | 10 |
| Silder et al. (2010b) | 1 | 1 | 1 | 0 | 1 | 1 | 1 | 1 | 1 | 1 | 9 |
| Evangelidis et al. (2023) | 1 | 0 | 0 | 1 | 1 | 1 | 1 | 1 | 1 | 1 | 8 |
| Yamashita et al. (2023) | 1 | 1 | 0 | 1 | 0 | 1 | 1 | 1 | 0 | 1 | 7 |
| Miyamoto et al. (2024) | 1 | 1 | 0 | 1 | 1 | 1 | 1 | 1 | 1 | 1 | 9 |

Ten-item criteria: (1) clarity of research questions and hypotheses; (2) appropriateness of participants and sampling; (3) rigor of experimental design; (4) reliability of measurement methods and instruments; (5) adequacy of data processing and statistical analysis; (6) completeness of results presentation; (7) depth of result interpretation and discussion; (8) ethical compliance and transparency; (9) ecological and external validity; (10) practical value and translational significance
